# Medial clavicle pseudarthrosis successfully treated with an inverted distal clavicle locking plate

**DOI:** 10.1016/j.amsu.2019.06.002

**Published:** 2019-06-06

**Authors:** Yu Sasaki, Sang Yang Lee, Takashi Iwakura, Tomoaki Fukui, Keisuke Oe, Tomoyuki Matsumoto, Takehiko Matsushita, Teruya Kawamoto, Yutaka Mifune, Ryosuke Kuroda, Takahiro Niikura

**Affiliations:** Department of Orthopaedic Surgery, Kobe University Graduate School of Medicine, 7-5-1 Kusunoki-cho, Chuo-ku, Kobe, 650-0017, Japan

**Keywords:** Medial clavicle, Pseudarthrosis, Open reduction, Locking plate fixation

## Abstract

**Introduction:**

Medial clavicle fractures are rare injuries. Symptomatic nonunion arises up to 8% of medial clavicle fractures when treated conservatively.

**Presentation of case:**

A 53-year-old man sustained a left medial clavicle fracture and was treated conservatively at another hospital. Nine months after his initial injury, he was referred to our institution. We diagnosed pseudarthrosis of the medial clavicle. We performed open reduction and internal fixation using an inverted distal clavicle locking plate. At the 1-year follow-up, radiographs showed bone union.

**Discussion:**

This is the first reported case of medial clavicle pseudarthrosis treated with an inverted distal clavicle anatomical locking plate. There are several advantages in using this plate.

**Conclusion:**

This method is a good treatment option.

## Highlights

• Medial clavicle pseudarthrosis is very rare.
• The optimal fixation device for the medial clavicle has not yet been established.
• We first used an inverted distal clavicle anatomical locking plate to treat it.
• This method is a good treatment option.

## 1 Introduction

Medial clavicle fractures are rare injuries, accounting for only 2%–3% of all clavicle fractures [[1], [2], [3], [4]]. Traditionally, these fractures have been managed conservatively, even when significantly displaced [5]. However, symptomatic nonunion arises in approximately 8% of medial clavicle fractures when treated conservatively [6]. Few reports have addressed the treatment of medial clavicular nonunion because of the rarity of this fracture. We herein present a rare case of medial clavicle pseudarthrosis that was successfully treated with an inverted distal clavicle locking plate. This work has been reported in line with the SCARE 2018 criteria [7].

## 2 Presentation of case

A 53-year-old male construction worker fell from the second floor of a house while working and sustained an injury to his left shoulder. He was diagnosed with a left medial clavicle fracture (AO/OTA classification: 15-A1, Robinson classification: Type 1B) and was treated conservatively with a figure-of-eight bandage at another hospital. Nine months after his initial injury, he was referred to our institution with complaints of abnormal mobility, pain, and activity limitation.

Physical examination revealed tenderness, deformity and abnormal mobility at the medial aspect of his left clavicle. He also had limited range of motion in his left shoulder (130° of flexion and 110° of abduction). There were no signs of dysphagia or dyspnea. The Japanese Society for Surgery of the Hand Version of the Disability of the Arm, Shoulder, and Hand (DASH-JSSH) score was 46 points for disability/symptom subscale and 50 points for work subscale. He had no surgical history, drug history, family history including any relevant genetic information, and psychosocial history.

**Fig. 1:**
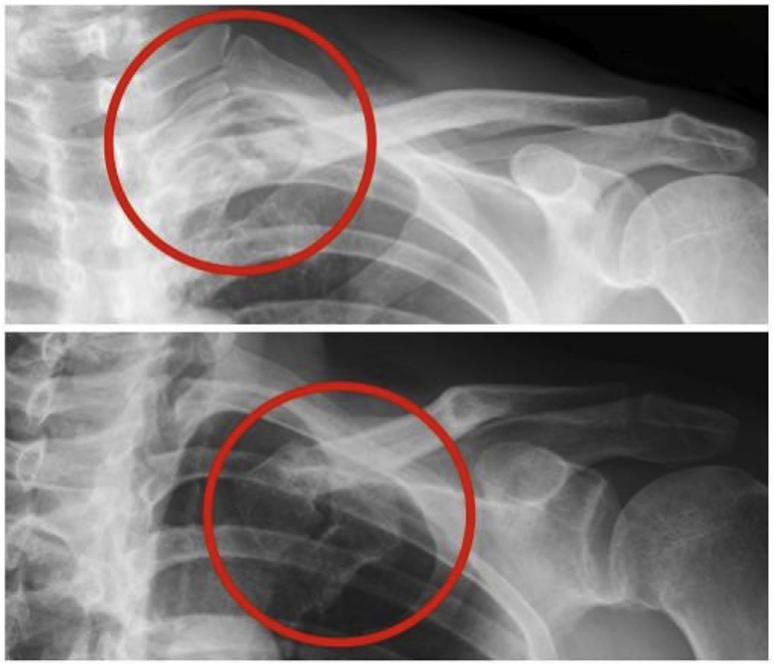
Preoperative radiographs of the left-sided medial clavicle fracture. The red circles indicate the fracture site.

**Fig. 2:**
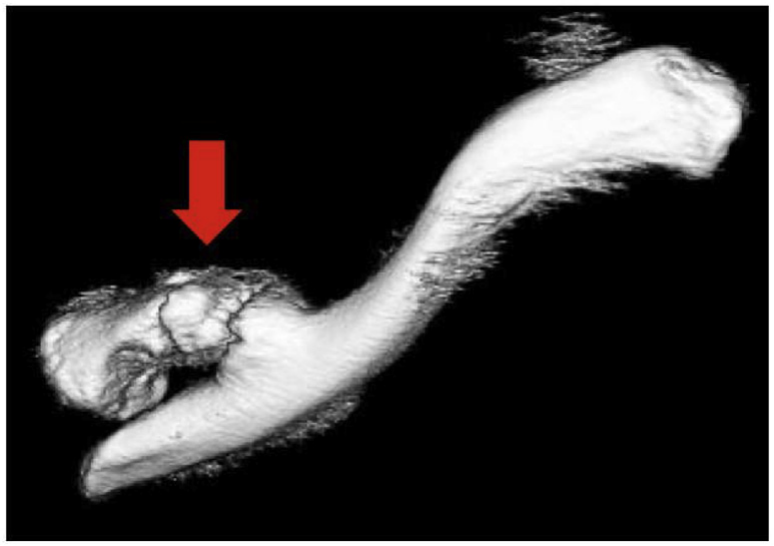
Three-dimensional computed tomography reconstruction demonstrating shortening and displacement of left medial clavicle pseudarthrosis (red arrow).

Radiographs taken in our institution 9 months after his initial injury showed a medial clavicle fracture with displacement and deformity of the medial clavicle (Fig. 1. 1). A computed tomography (CT) scan including three-dimensional reconstruction revealed further details: an oblique extra-articular fracture of the medial clavicle with significant displacement, deformity, and shortening of the clavicle (Fig. 2). The lateral fragment was displaced anteriorly, and the fracture site was not united. We diagnosed symptomatic pseudarthrosis of the medial clavicle.

**Fig. 3:**
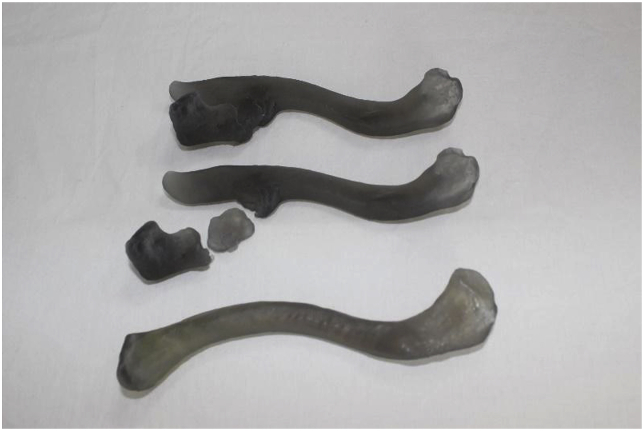
Three-dimensional models created from computed tomography scan data. The two models at the top are the affected-side clavicles. The model at the bottom is the mirror image of the contralateral clavicle.

Surgery was planned because the patient had severe pain with activity limitation and hyper-mobile pseudarthrosis. For preoperative planning, the life-size three-dimensional model [8] of the mirror image of the contralateral clavicle was created from the CT scan data to select the most suitable implant (Fig. 3). An inverted ipsilateral locking plate originally designed for the distal clavicle (locking compression plate [LCP] or LCP clavicle plate with lateral extension; DePuySynthes, Raynham, MA, USA) fit the model well.

**Fig. 4:**
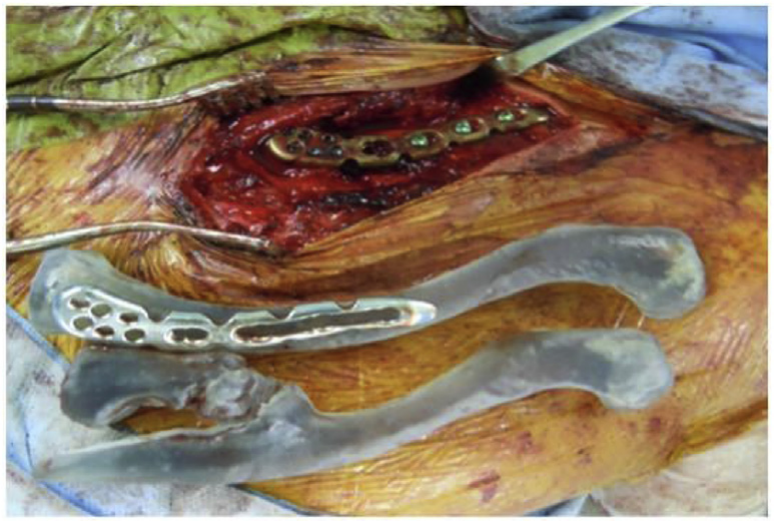
Intraoperative photograph showing exact matching of the distal clavicle anatomic locking plate.

We performed open reduction with debridement of the pseudarthrotic tissue and internal fixation using the locking plate. Surgery was performed 11 months post-injury. The patient was placed in the beach chair position under general anesthesia. A direct curved approach over the medial clavicle was made. The bony bulge of the lateral fragment was then excised with a bone saw to expose the pseudarthrotic region. The pseudocapsule and synovial fluid were recognized between the medial and lateral fragments. After removal of the pseudarthrotic tissue and anatomical reduction of the fragments, the previously mentioned inverted locking plate was placed on the anterosuperior aspect of the medial clavicle. It exactly matched the medial clavicle with no contouring (Fig. 4). The medial fragment was fixed with five 2.7-mm locking head screws, and the lateral fragment was fixed with three 3.5-mm locking head screws. Finally, the resected bony bulge was broken down into small pieces and grafted into the pseudarthrotic site.

**Fig. 5:**
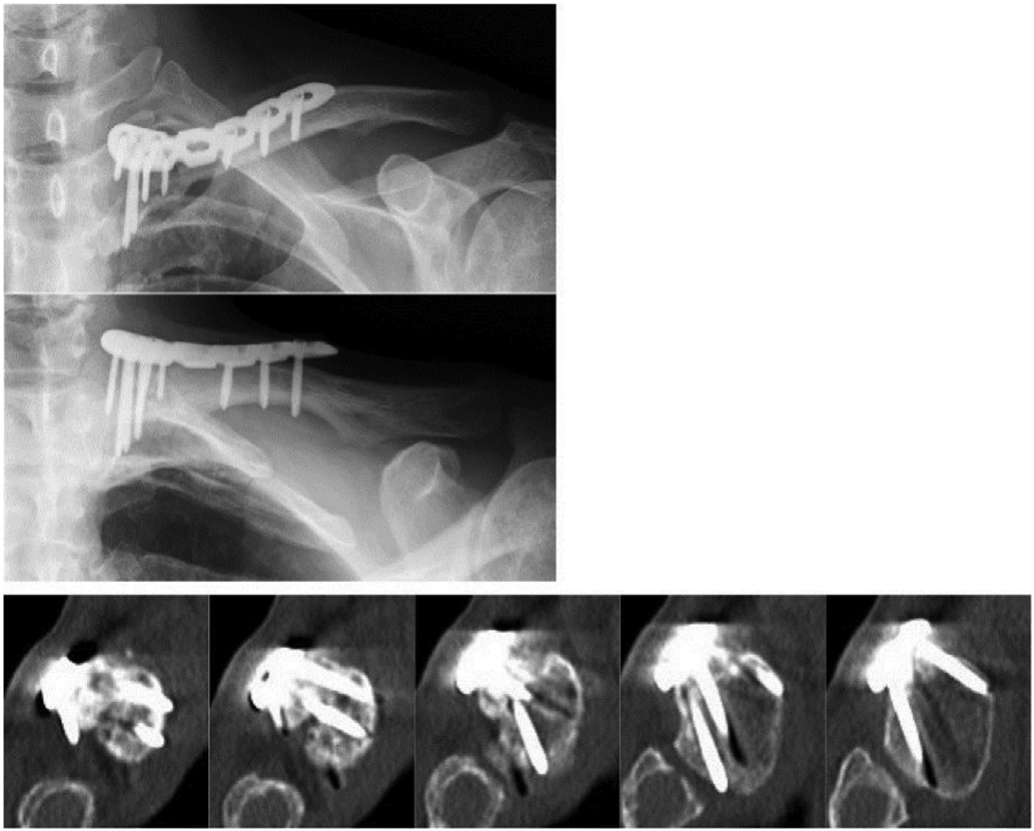
One-year follow-up radiographs demonstrating union of the fracture without loosening of the internal fixation. A computed tomography scan showed that all 2.7-mm locking head screws had been precisely and safely inserted into the medial fragment.

After the surgery, the bony bulge and abnormal mobility at the patient's left clavicle had disappeared. No complications such as neurovascular injury or surgical site infection occurred during the perioperative period. The affected upper limb was immobilized in a sling for 5 weeks postoperatively. The patient was encouraged to start functional exercises after the removal of the sling. At the 1-year follow-up, radiographs showed bone union without loosening of the internal fixation. A CT scan showed that all 2.7-mm locking head screws were inserted in the medial fragment exactly and safely (Fig. 5). He reported no pain and had regained almost the full range of motion of his affected shoulder joint. He was able to return to his pre-injury occupational and activity levels. The DASH-JSSH score improved to 9.17 points for the disability/symptom subscale and 25 points for work subscale at 1 year after the surgery.

## 3 Discussion

Medial clavicle fractures are uncommon. Most of these fractures can be treated nonoperatively with an immobilizing sling or brace [2]. The indications for surgical repair of medial clavicle fractures have traditionally included open fractures, neurovascular involvement, or a threat to the integrity of the overlying skin, even in the presence of significant displacement [5].

Some recent studies have recommended open reduction and internal fixation for displaced or comminuted medial clavicle fractures to prevent nonunion and dysfunction, especially in young or physically active individuals [2,9,10]. A few techniques have been described for surgical reduction and stabilization of these injuries, including wire or plate fixation. However, K-wire fixation has been proven unsafe because of breakage and migration. According to previous reports, fixation with K-wires occasionally results in injuries to the lungs, esophagus, major vessels, or spinal cord [11]. Oe et al. [2] recommended the use of small fragment locking plates. The authors concluded that these plates had advantages including decreased loss of reduction secondary to screw toggling and higher stability during early motion. Moreover, Tokiyoshi [12], Wang et al. [13] and Titchener et al. [14] reported the use of an inverted distal clavicle anatomic locking plate for acute medial clavicle fractures. They showed that this plating method allowed for safe rigid fixation.

In another study, however, 8.3% of the patients treated nonoperatively developed nonunion [6]. Some reports have shown that clavicle nonunion resulted in pain or subjective impairment persisting long after the initial trauma [15,16]. In the present case, the patient developed persistent pain in his left medial clavicle and limited range of motion in his left shoulder. Therefore, we diagnosed him with pseudarthrosis and decided to perform surgery.

Few reports have mentioned the surgical treatment of medial clavicle nonunion. Only three case reports to date have described such treatment. Der Tavitian et al. [17] reported a case of medial clavicle nonunion that healed after internal fixation with a lag screw and bone graft. Al-Yassari et al. [18] recommended two-stage surgical treatment involving tension band wiring and fixation with a dynamic compression plate after removal of the tension band wiring. They stated that the transferred stresses and motion from shoulder movement to the medial clavicle and sternoclavicular joint were the causes of device migration, particularly of the K-wires, and resultant repair failure. The two-stage approach offloaded the medial clavicle area during the healing process. Kim et al. [19] described a surgical procedure using a small T-shaped plate for the distal radius and multiple tension band sutures. They proposed the use of tension band sutures to prevent fragment distraction and screw pull-out between the medial fragment of the clavicle and the T-shaped plate.

In the present case, we used an inverted ipsilateral locking plate that was originally designed for distal clavicle fractures. This is because preoperatively, we found that the plate fit the clavicle using a life-size three-dimensional model of the mirror image of the contralateral clavicle [8]. In fact, the inverted plate matched the anatomically reduced medial clavicle well with no contouring. To our knowledge, this is the first reported case of medial clavicle pseudarthrosis treated with an inverted distal clavicle anatomical locking plate.

We conclude that there are several advantages in using this plate. First, the plate matches the clavicle well with no contouring. Second, the plate is thin; its maximum thickness is only 3 mm. Thus, soft tissue irritation is minimal. Finally, multiple 2.7-mm locking head screws that are inserted in the small medial fragment can achieve rigid fixation. This contributes to early functional exercise, decreases the risk of nonunion, and supports fracture healing.

## 4 Conclusion

Medial clavicle pseudarthrosis was successfully treated with an inverted ipsilateral locking plate for the distal clavicle. Although the optimal fixation device for the medial clavicle has not yet been established, we believe that the herein described method is a good treatment option.

## Ethical approval

Approval to publish this case report was waived by the institution.

## Sources of funding

This research did not receive any specific grant from funding agencies in the public, commercial, or not-for-profit sectors.

## Author contribution

All authors in this manuscript contributed to the interpretation of data, and drafting and writing of this manuscript. Yu Sasaki is first author and Takahiro Niikura is corresponding author of this paper. They and San Yang Lee, Takashi Iwakura, Tomoaki Fukui, Keisuke Oe, Tomoyuki Matsumoto, Takehiko Matsushita, Teruya Kawamoto, Yutaka Mifune conceived and designed the study and drafted the manuscript. Ryosuke Kuroda is the chief of orthopaedic surgery in our university hospital. All the authors read and approved the final manuscript.

## Conflicts of interest

None.

## Research registration number

Not applicable.

## Guarantor

Takahiro Niikura.

## Consent

We got oral consent from the patient for publication of this case report.

We do not have signed consent from the patient unfortunately because the patient stopped attending our institution without our permission.

We have tried to contact with the patient and his family many times so as to get the consent document. However, he did not come to our hospital again.

Because we don't have signed consent, the head of our medical team is sure to take responsibility that exhaustive attempts have been made to contact with the family and that the paper has been sufficiently anonymised not to cause harm to the patient or their family.

## Provenance and peer review

Not commissioned externally peer reviewed.

## Appendix A Supplementary data

scare_2018_guidelines.docx scare_2018_guidelines.docx
